# Supplementary material for: Evaluation of the Thermal Response of the Horns in Dairy Cattle
Source: Animals (Basel). 2023 Jan 31;13(3):500. doi: 10.3390/ani13030500 (PMC9913495; doi:10.3390/ani13030500)
Supplement: Supplementary file 1 [file animals-13-00500-s001.zip › animals-2144787 supplementary materials.pdf]

animals MDPI

animals-2144787 Supplementary materials

Table S1. An overview of the focal cows observed during the first part of this study (horn temperature in relation to behaviour and environment). As for the breeds: FH = German Black Pied (Fries-Hollands), HF = Holstein Friesian, MRY = Meuse-Rhine-Yssel, UNK = unknown. Farm A and B: horned cows, Farm C: hornless cows.

Farm	Cow ID	Date of birth	Date of giving birth before observations	Breed	Coat colour
A	A1	17/12/2018	25/02/2021	62% MRY 25% UNK	Red
	A2	15/09/2017	23/06/2020	100% FH	Black
	A3	13/10/2016	24/03/2021	87% MRY, 12% HF	Red
	A4	12/11/2015	23/05/2020	50% FH, 50% UNK	Black
	A5	30/08/2014	04/06/2020	75% MRY, 25% HF	Red
	A6	21/02/2011	28/05/2020	50% FH, 37% UNK, 12% HF	Black
B	B1	12/10/2018	10/11/2020	Jersey	Beige
	B2	24/12/2017	07/02/2020	Jersey	Beige
	B3	02/09/2016	11/10/2019	Jersey	Beige
	B4	04/07/2016	05/11/2020	Jersey	Beige
	B5	31/05/2013	25/12/2019	Jersey	Beige
	B6	08/05/2012	12/12/2020	Jersey	Beige
C	C1	01/03/2019	21/02/2021	HF	Black
	C2	10/09/2018	29/09/2020	HF	Black
	C3	12/12/2016	30/09/2020	HF	Black
	C4	13/06/2016	28/10/2020	HF	Black
	C5	20/03/2014	02/02/2021	HF	Black
	C6	27/05/2013	10/11/2019	HF	Black

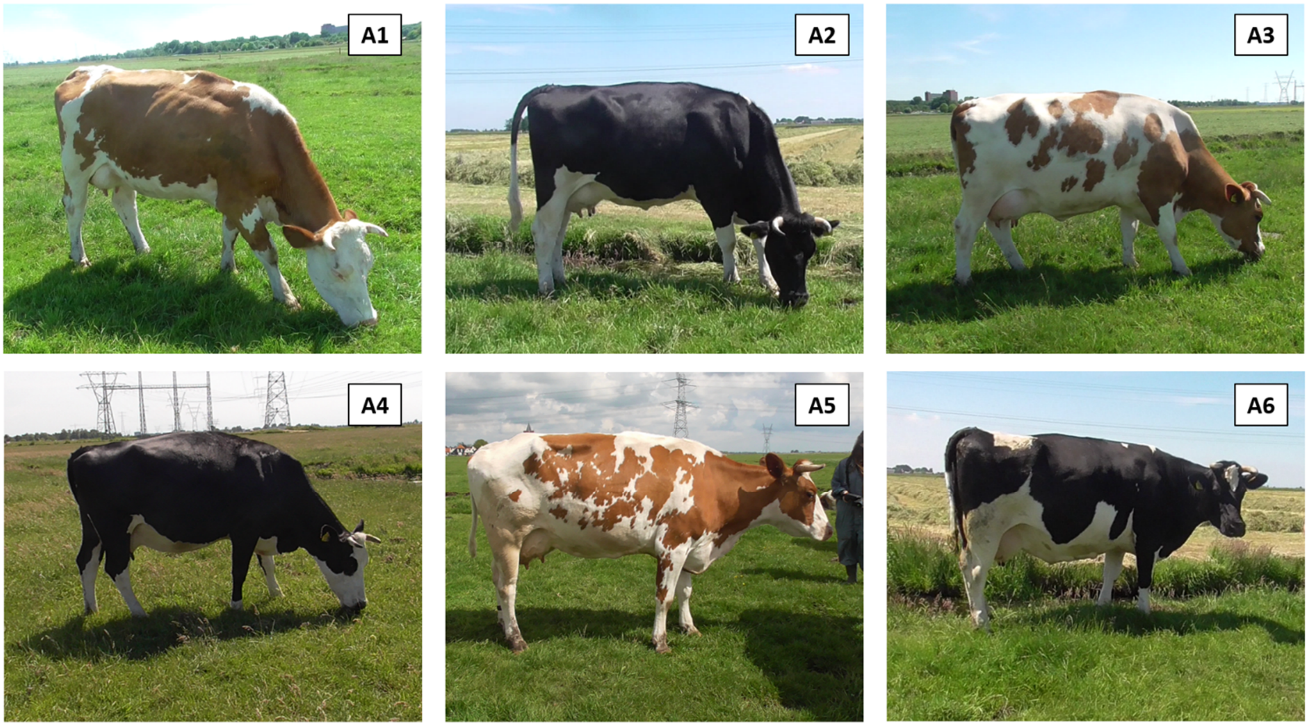

Figure S1. Images of the focal cows observed on farm A during the first part of this study (horn temperature in relation to behaviour and environment).

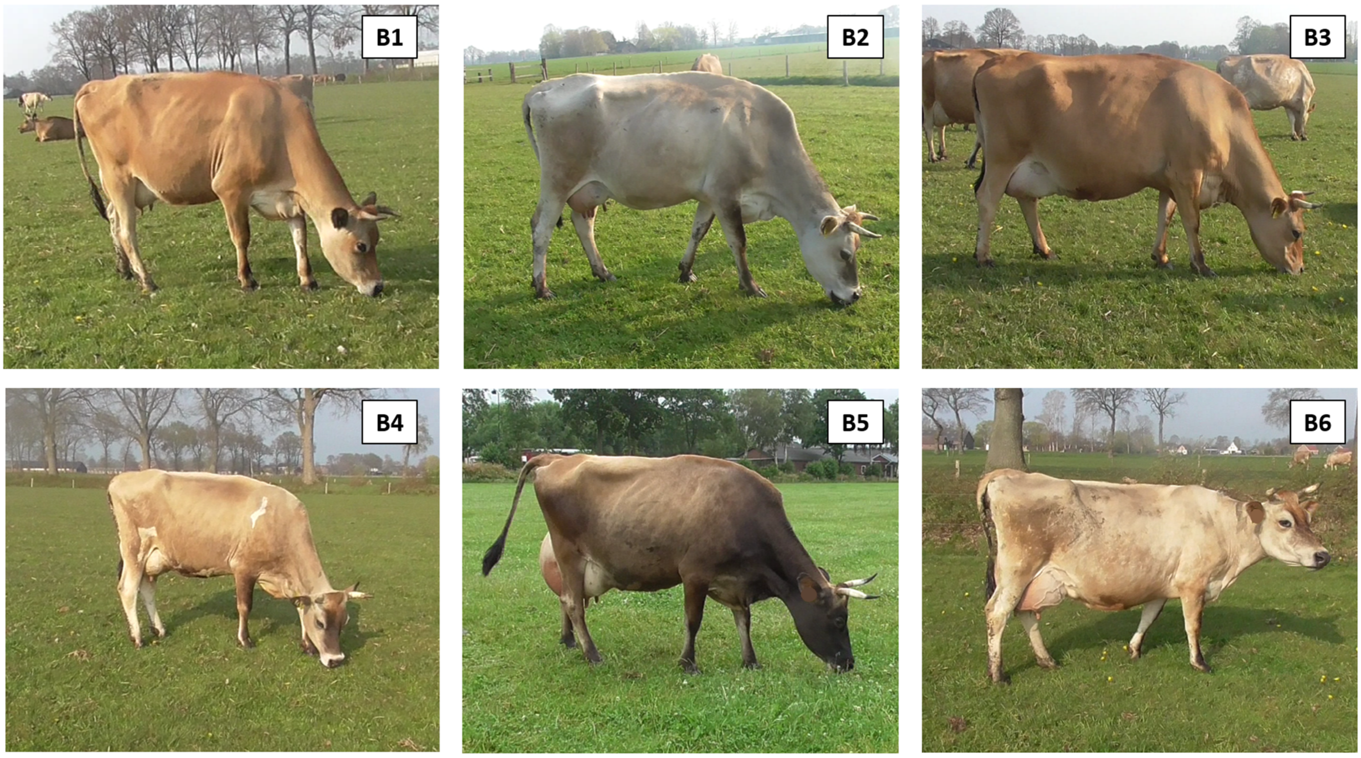

Figure S2. Images of the focal cows observed on farm B during the first part of this study (horn temperature in relation to behaviour and environment).

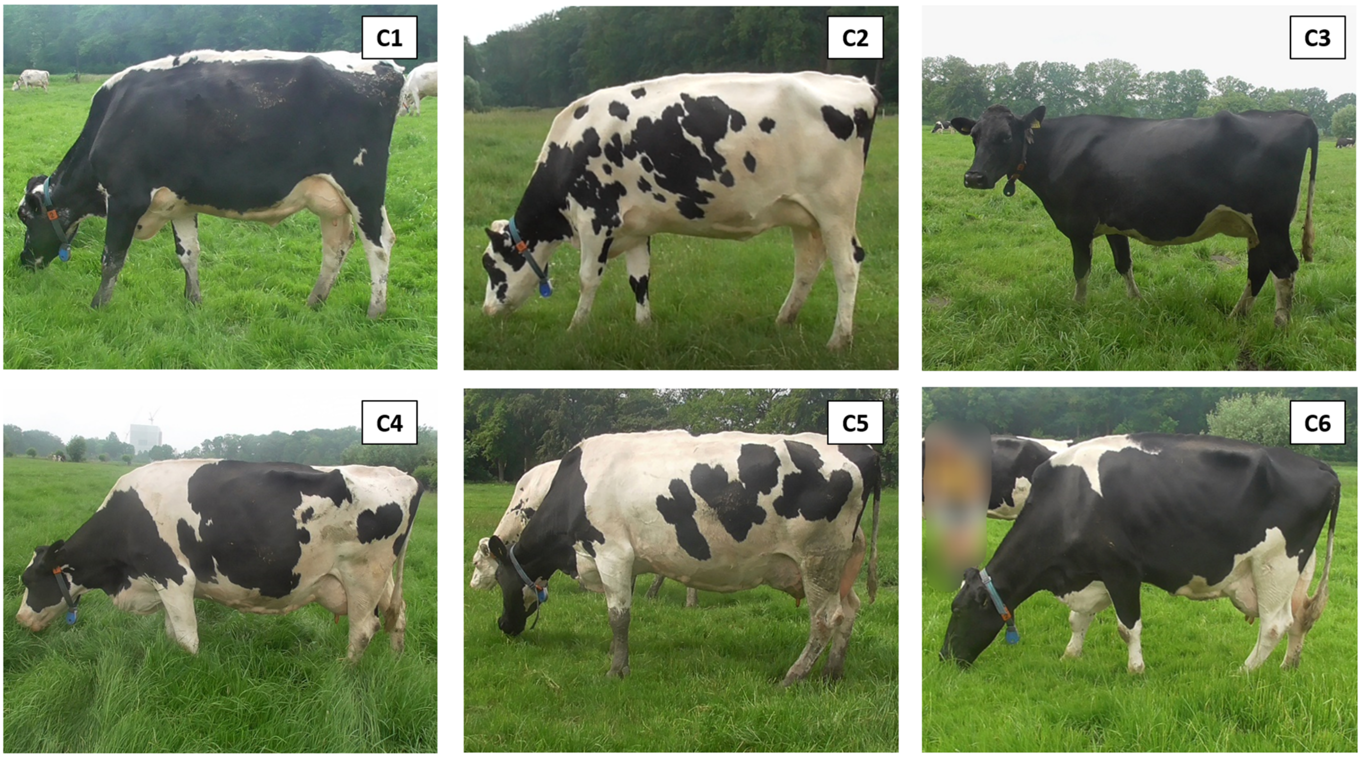

Figure S3. Images of the focal cows observed on farm C during the first part of this study (horn temperature in relation to behaviour and environment).

Protocol S1: Detailed procedures for data collection and processing.

Data collection - behaviour observations and thermal imaging

1. Before starting the observation, the thermal camera was allowed to warm up (ca. 5 minutes).
2. The thermal camera was held horizontally to the focal individual, at eye level, at an angle between 90º and 45º (ensuring the eye and ear were visible from the front) and at a distance to the head of the cow of around 1 to 1,5 meters. See Figure S4. for examples of acceptable angles.

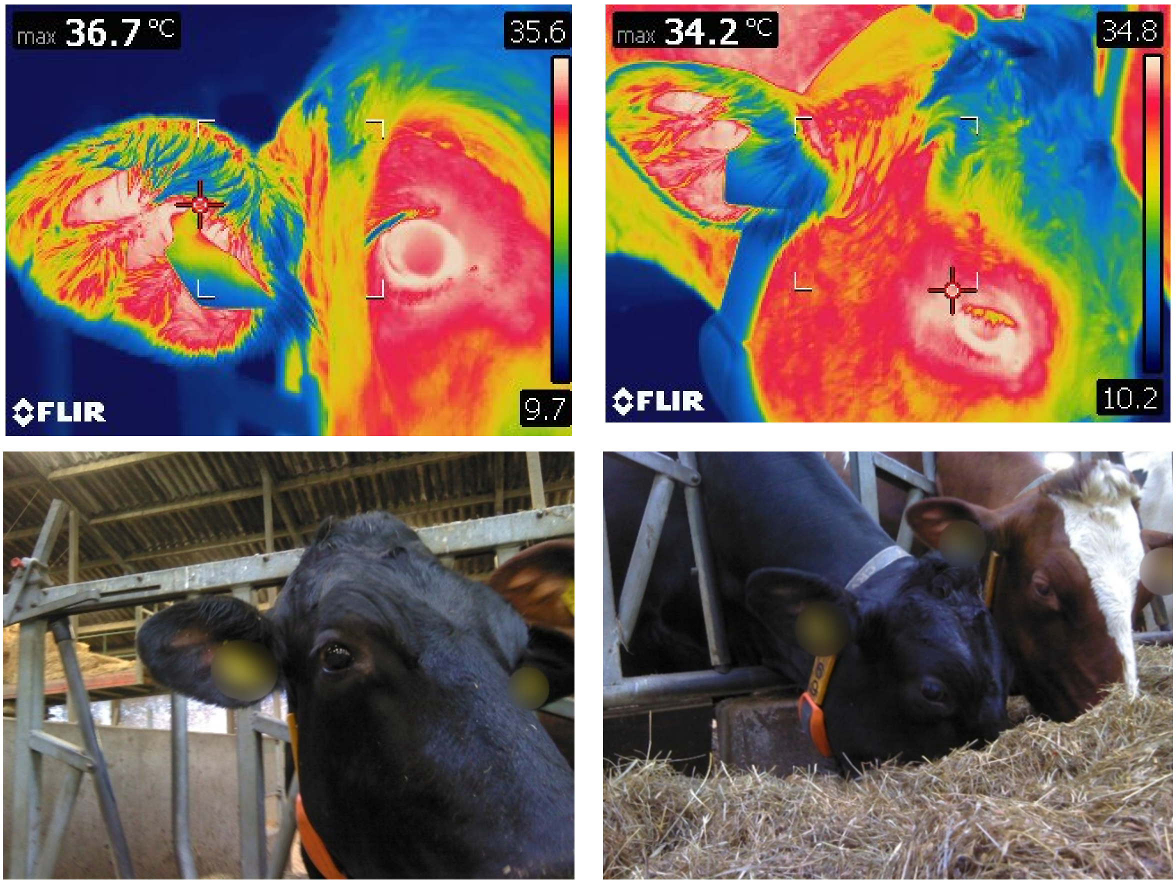

Figure S4. Examples of acceptable angles for the thermal images. The two pictures on the top show the IRT images as would be shown on camera, and the two images on the bottom are the same images but in normal non-IRT format.

3. The second observer held the camcorder horizontally to the cow, at a distance to the cow of around 4-5 meters, so that the cow, as well as a diameter of approximately two meters around her, was entirely visible, with the height of camcorder more or less at the same height of the cow.
4. The Kestrel was hung on the camcorder to avoid direct contact with the observer influencing the environmental parameters.
5. During the first 30 seconds of recording, information about the observation was mentioned in this order: day and time (in seconds) at the moment of starting the video, air temperature, air humidity, wind speed, wind direction, location, and the number of the cow that was being observed (number on the ear tags of the cow).
6. Start observations and thermal imaging. All behaviours were mentioned and as many infrared pictures as possible taken, alternating between the left and right side of the head of the cow – first 2 pictures on one side, then 2 on the other side.
7. If the cow was out of sight for more than 5 minutes, the observation was stopped and re-started once the cow was in sight again.

8. After 5 minutes, the observation was completed

Data processing

Setting up FLIR ResearchIR for IRT image analyses

9. In accordance with previous studies regarding IRT measurements in cattle, the following data were used:
   a. Emissivity: 0.98 [1–7].
   b. Reflected temperature: 20 ºC [8,9].
   c. Distance: 1 m (or another distance if for that observation it was necessary to stand farther away).
10. Then create functions for the regions of interest (ROI) of the study (horns, eyes and ears).

Analysing IRT images in FLIR ResearchIR

11. Load all IRT images of one observation day into the program
12. Incorrect images are excluded from analysis. An image is incorrect if:
    a. It is too blurry.
    b. If the ROI is not clearly visible.
       i. For the ROI "ear", images are excluded if the tag was not visible and/or only half or less than half of the inside of the ear was visible (see Figure S5).

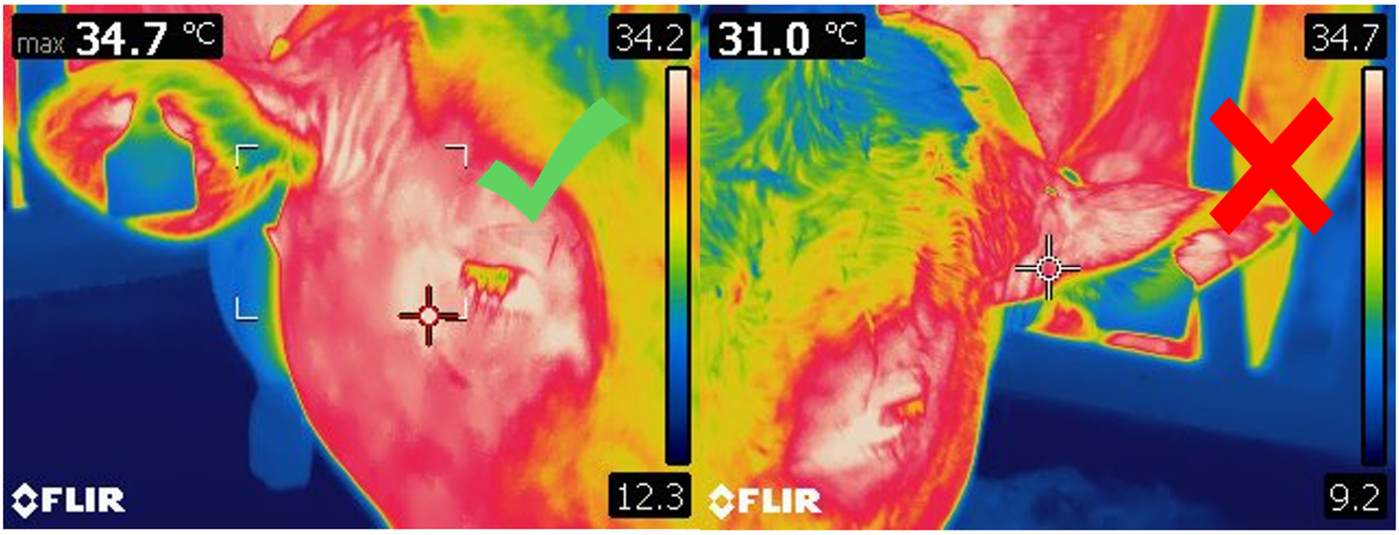

Figure S5. Thermal images of ears. The left image shows a correct thermal image included for analysis: the entire outline of the ear tag and more than half of the inside ear is clearly visible. The right image shows an excluded as the entire outline of the tag is not visible and less than half of the inside ear is visible.

       ii. For the ROI "eye", images are excluded if the lachrymal region of the eye is not visible (see Figure S6).

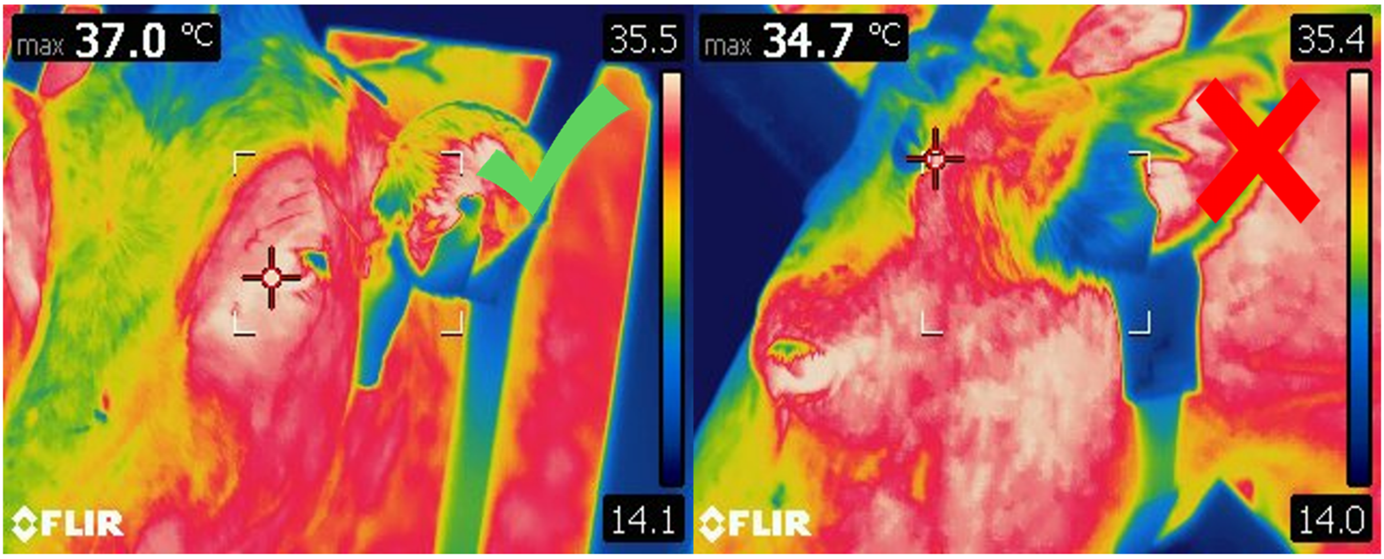

Figure S6. Thermal images of eyes. The left image shows a correct thermal image included for analysis, as the lachrymal region is visible from the front. The right image shows an excluded image as the lachrymal region is not in sight.

iii. For the ROI "horn", images are excluded if the base of the horn is not visible (see Figure S7).

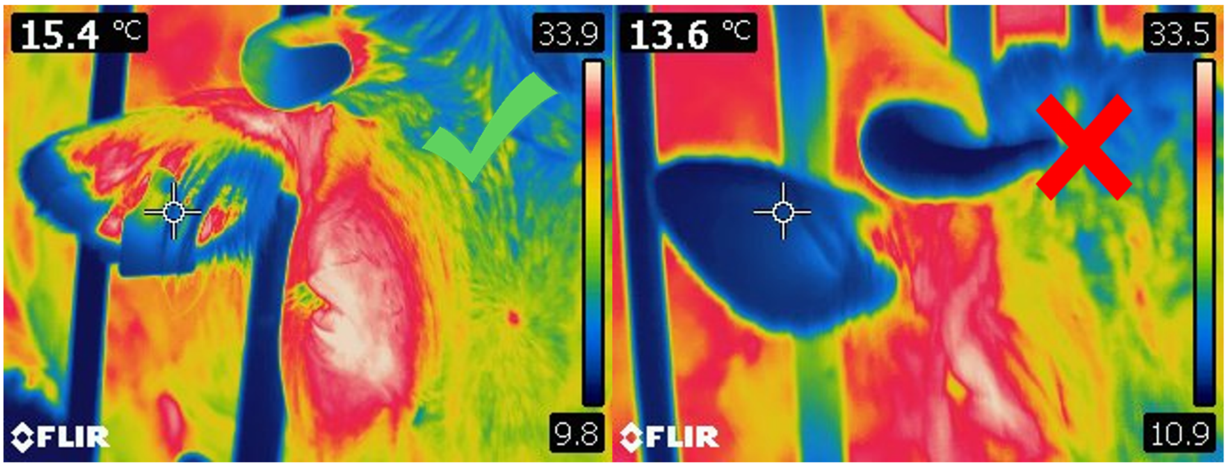

Figure S7. Thermal images of horns. The left image shows a correct thermal image included for analysis, as the horn base is visible from the front. The right image shows an excluded image, as the horn base is not clearly visible due to obstruction of the tip of the horn.

c. If at least one of the ROIs was clearly visible and at the correct angle, then the image was used for analysis, excluding the region(s) that were not clearly visible and at the correct angle.

13. Only one of duplicate images (images that look very similar and are taken only a few seconds apart) is included in the analyses (the higher quality image, i.e., the least blurry and with the most correctly visible regions).
14. For each included image, the atmospheric temperature and humidity of the corresponding observation are entered into the program.
15. To analyse the included images, the horns, eyes and ears of the cow are selected using the regions of interest (ROIs) function. The ROI always encompassed the warmest spot of the corresponding area (Figure S8).

d. For the ears and horns, the polygon ROI is used, covering the entire ear inside and horn.

e. For the eye area, the ellipse ROI is used, surrounding the entire eye, eyelid, lachrymal region, and a narrow band of skin around the eye (around 1 cm width). Note that with very high environmental temperature a smaller ROI was applied to avoid the interference of hair surroinding the eye region).

16. Following the definition of the ROIs, the temperature data of the area in the ROIs is shown using the 'Statistics Viewer'. The data can then be saved via the save button in the functions section.
17. Export the temperature data per ROI to a .txt file

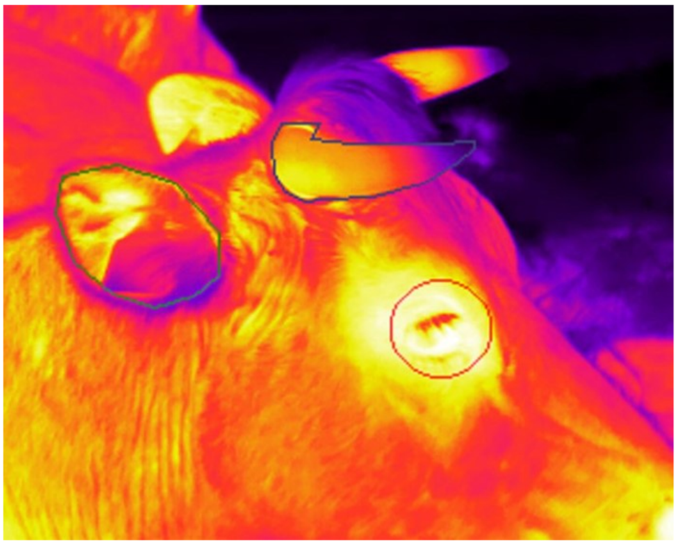

Figure S8. Thermal image example of ROIs for the three studied areas eye, ear, horn.

18. After having analysed all images of one observation day, the individual .txt files of each analysed image are loaded into R. Use script to create Excel file with all the minimum, mean and maximum temperatures of the ROIs.

Horn temperature in relation to rumination

The protocol followed the same procedures as described for Part 1, except that thermal images were taken of the front of the cows (Figure S9).

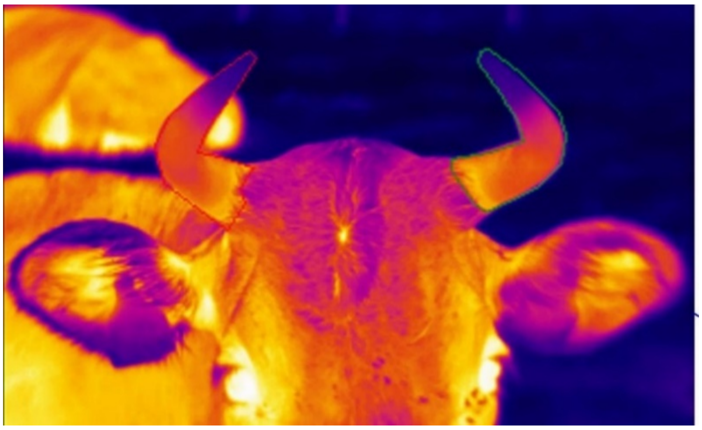

Figure S9. Thermal image example of the position of the cow to study horn temperature in relation to rumination.

Script S1: R script for data preparation

Extracting the times of the thermal photos

For gathering the data of the temperatures and the behaviours in one datasheet, it was first necessary to extract the exact times at which the photos were made. The following script will do this.

First set the variable 'date' to the correct date.

```
date <- "20210504"
FLIRfolder <- paste0("Example data/", date)
```

Then make a dataframe with the names of the photos and the times at which they were taken:

```
Photos = list.files(path = FLIRfolder)
PhotosDf <- data.frame(Photos)
DateTimes <- lapply(paste(FLIRfolder, Photos, sep = "/"), FUN = file.mtime)
DateTimes <- Reduce(c, DateTimes)
Time <- format(DateTimes, format = "%H:%M:%S")
PhotoTimesDf <- data.frame(PhotosDf, Time)

library(writexl)
write.csv(PhotoTimesDf, paste0("Example data\\PhotoTimes ", date, ".csv"))
```

Matching behaviours to the thermal photos

After the behavioural observations, the behaviours were gathered into timelogs in excel. The following script made sure the correct behaviours were matched to each FLIR photo.

First, a new phototimes document was made that only containeds the pictures that were actually used in the analyses.

```
library(readxl)
PhotoTimes <- data.frame(read_excel(paste0("Example data/PhotoTimes ",
                                            date,
                                            " final.xlsx")))
```

In the thermocamera, time ran more quickly than in real life, causing the phototimes not to match the actual times. When the observers found out about this, it was checked for each day how much difference there was between the camera time and the real time by taking a picture of the exact time with the camera. Based on this, the differences of the days before were also calculated.

```
PhotoTimes$TimeNum <- as.numeric(PhotoTimes$Time)

Delay <- 78

PhotoTimes$ActualTimeNum <- PhotoTimes$TimeNum - Delay
PhotoTimes$ActualTime <- as.POSIXct(PhotoTimes$ActualTimeNum,
                                    origin = "1970-01-01", tz = "GMT")
```

Next, import the timelog of the behaviours for the corresponding day:

```
Timelog <- data.frame(read_excel(paste0("Example data/Behaviour timelog ",
                                         date,
                                         ".xlsx")))
```

```
StarttimesDur <- Timelog$Time.start
EndtimesDur <- Timelog$Time.end
n <- nrow(PhotoTimes)
NAvector <- rep(NA, length.out = n)
zeroVector <- rep(0, length.out = n)
Round <- NAvector
Farm <- NAvector
CowNr <- NAvector
Column1 <- NAvector
Column2 <- NAvector
ObsIRT <- NAvector
ObsBeh <- NAvector
BP <- NAvector
SC <- NAvector
DurationNS <- NAvector
DurationS <- NAvector
Location <- NAvector
Wind_dir <- NAvector
Temperature <- NAvector
Humidity <- NAvector
WindSp <- NAvector
Side <- NAvector
EyeTemp <- NAvector
EarTemp <- NAvector
HornTemp <- NAvector
Remarks <- NAvector

AllData <- data.frame(PhotoTime = PhotoTimes$Time,
                      PhotoActualTime = PhotoTimes$ActualTime,
                      Round = Round,
                      PhotoName = PhotoTimes$Name,
                      Farm = Farm,
                      CowNr = CowNr,
                      Column1 = Column1,
                      Column2 = Column2,
                      ObsIRT = ObsIRT,
                      ObsBeh = ObsBeh,
                      BP = BP,
                      SC = SC,
                      DurationNS = DurationNS,
                      DurationS = DurationS,
                      Location = Location,
                      Wind_dir = Wind_dir,
                      Temperature = Temperature,
                      Humidity = Humidity,
                      WindSp = WindSp,
                      Side = Side,
                      EyeTemp = EyeTemp,
                      EarTemp = EarTemp,
                      HornTemp = HornTemp,
                      Remarks = Remarks)

for(j in 1:nrow(PhotoTimes)){
  t <- PhotoTimes[j,5]
  for(i in 1:nrow(Timelog)){
    if(t <= EndtimesDur[i] & t >= StarttimesDur[i]){
      AllData$Round[j] <- Timelog[i, 3]
```

```
        AllData$Farm[j] <- Timelog[i, 2]
        AllData$CowNr[j] <- Timelog[i, 4]
        AllData$ObsIRT[j] <- Timelog[i, 19]
        AllData$ObsBeh[j] <- Timelog[i, 20]
        AllData$BP[j]<- Timelog[i,9]
        AllData$SC[j]<- Timelog[i,10]
        AllData$DurationNS[j] <- Timelog[i,11]
        AllData$DurationS[j] <- Timelog[i,12]
        AllData$Location[j] <- Timelog[i, 13]
        AllData$Wind_dir[j] <- Timelog[i, 14]
        AllData$Temperature[j] <- Timelog[i,16]
        AllData$Humidity[j] <- Timelog[i,17]
        AllData$WindSp[j] <- Timelog[i,18]
        AllData$Remarks[j] <- Timelog[i,21]}
  }
}
```

Extracting the thermal data

The thermal data was analysed using FLIR software. Per photo, a .txt file was made that contained the thermal parameters. The script below is for extracting the necessary data from these .txt files so they can be put into the master data sheet.

```
library("purrr")
library("tidyverse")
setwd(paste0("Example data/Txt ", date))
temp = list.files(pattern = ".txt")
myfiles = lapply(temp, read.delim, na = "N/A")
myfiles1 <- myfiles %>% reduce(left_join, by = "Name")
FLIRdata <- data.frame(myfiles1)

oddsMinOne <- seq(3,10000,2)
FLIRdata <- FLIRdata[,-oddsMinOne]

n <- FLIRdata$Name
FLIRdata <- as.data.frame(t(FLIRdata[,-1]))
colnames(FLIRdata) <- n
```

Script S2: R script for statistical analyses

```
library(dplyr)
library(EnvStats)
library(gridExtra)
library(lme4)
library(doBy)
library(ggplot2)
library(ggpubr)
library(grid)
library(pastecs)
library(readxl)
library(rmcorr)
```

To store a colorblind friendly colour palette of 12 colours:

```
ColPal <- c("#88CCEE", "#CC6677", "#DDCC77", "#117733", "#332288", "#AA4499",
            "#44AA99", "#999933", "#882255", "#661100", "#6699CC", "#888888")

Data <- read.csv("Data.csv")
```

Descriptive statistics

How many photos in total?

```
(n <- nrow(Data))

## [1] 8376
```

How many photos per cow?

```
Data$CowNr <- as.factor(Data$CowNr)
table(Data$CowNr)

##
##  A1  A2  A3  A4  A5  A6  B1  B2  B3  B4  B5  B6  C1  C2  C3  C4  C5  C6
## 524 529 476 536 531 536 364 188 415 377 425 339 505 557 500 507 534 533
```

Make a table of the basic descriptive statistics:

```
attach(Data)
Parameters <- cbind(TempEnv, Humidity, WindSp,
                    HornTemp, EyeTemp, EarTemp, HLI)
options(scipen = 100)
options(digits = 2)
(DataSummary <- stat.desc(Parameters, basic = F))

##              TempEnv Humidity WindSp HornTemp EyeTemp EarTemp    HLI
## median        15.900    47.00  1.400   32.817  36.544  35.298  50.67
## mean          16.118    48.98  1.666   32.049  36.450  33.722  55.11
## SE.mean        0.058     0.17  0.013    0.058   0.014   0.062   0.14
## CI.mean.0.95   0.113     0.34  0.026    0.114   0.027   0.121   0.28
## var           28.047   255.51  1.338   17.732   1.490  28.549 165.32
## std.dev        5.296    15.98  1.157    4.211   1.221   5.343  12.86
## coef.var       0.329     0.33  0.694    0.131   0.033   0.158   0.23
```

How many right and left photos?

```
table(Data$Side)

##
##    L    R
## 4215 4161
```

And how many per cow?

```
table(Data$CowNr, Data$Side)

##
##        L   R
##    A1 258 266
##    A2 268 261
##    A3 245 231
##    A4 264 272
##    A5 268 263
##    A6 271 265
##    B1 182 182
##    B2  92  96
##    B3 215 200
##    B4 197 180
##    B5 220 205
##    B6 169 170
##    C1 256 249
##    C2 283 274
##    C3 251 249
##    C4 245 262
##    C5 273 261
##    C6 258 275
```

What does the data for each farm look like?

```
MeansPerRoundHT <- aggregate(HornTemp ~ ID_round*Farm, Data, mean)
MeansPerRoundEYT <- aggregate(EyeTemp ~ ID_round*Farm, Data, mean)
MeansPerRoundEAT <- aggregate(EarTemp ~ ID_round*Farm, Data, mean)

MeansPerRoundEYTEAT <- merge(MeansPerRoundEYT, MeansPerRoundEAT, by = c("ID_round", "Farm"))

MeansPerRoundHT <- arrange(MeansPerRoundHT, ID_round)
MeansPerRoundEYTEAT <- arrange(MeansPerRoundEYTEAT, ID_round)

wilcox.test(HornTemp ~ Farm, data = MeansPerRoundHT)

##
##  Wilcoxon rank sum test with continuity correction
##
## data:  HornTemp by Farm
## W = 1154, p-value = 0.5
## alternative hypothesis: true location shift is not equal to 0

kruskal.test(EyeTemp ~ Farm, data = MeansPerRoundEYT)

##
##  Kruskal-Wallis rank sum test
##
## data:  EyeTemp by Farm
## Kruskal-Wallis chi-squared = 39, df = 2, p-value = 0.000000004
```

```
kruskal.test(EarTemp ~ Farm, data = MeansPerRoundEAT)

##
##    Kruskal-Wallis rank sum test
##
## data:    EarTemp by Farm
## Kruskal-Wallis chi-squared = 3, df = 2, p-value = 0.3

dunnTest(EyeTemp ~ Farm,
         data=MeansPerRoundEYT,
         method="bonferroni")

## Warning: Farm was coerced to a factor.

## Dunn (1964) Kruskal-Wallis multiple comparison

##      p-values adjusted with the Bonferroni method.

##      Comparison     Z          P.unadj            P.adj
## 1          A - B    2.0 0.0490693201 0.1472079603
## 2          A - C -4.1 0.0000374915 0.0001124745
## 3          B - C -6.1 0.0000000011 0.0000000034

HornTempFarm <- ggplot(MeansPerRoundHT, aes(x = Farm, y = HornTemp, col = Farm)) +
  geom_boxplot(outlier.size=2,
               size = 1) +
  labs(x="Farm", y="Horn temperature (ºC)") +
  theme_bw(base_size = 25) +
  scale_color_manual(values = ColPal) +
  guides(col = "none") +
  coord_cartesian(ylim=c(14, 40))

EyeTempFarm <- ggplot(MeansPerRoundEYTEAT, aes(x = Farm, y = EyeTemp, col = Farm))    +
  geom_boxplot(outlier.size=2,
               size = 1) +
  labs(x="Farm", y="Eye temperature (ºC)") +
  theme_bw(base_size = 25) +
  scale_color_manual(values = ColPal) +
  guides(col = "none") +
  coord_cartesian(ylim=c(14, 40)) +
  scale_x_discrete(labels=c("A", "B", "C")) +
  geom_signif(comparisons = list(c("B", "C"),
                                 c("A", "C")),
              map_signif_level = TRUE,
              y_position = c(38, 39.5),
              size = 1,
              textsize = 8)

EarTempFarm <- ggplot(MeansPerRoundEYTEAT, aes(x = Farm, y = EarTemp, col = Farm))    +
  geom_boxplot(outlier.size=2,
               size = 1) +
  labs(x="Farm", y="Ear temperature (ºC)") +
  theme_bw(base_size = 25) +
  scale_color_manual(values = ColPal) +
  guides(col = "none") +
  coord_cartesian(ylim=c(14, 40)) +
  scale_x_discrete(labels=c("A", "B", "C"))
```

```
grid.arrange(HornTempFarm, EyeTempFarm, EarTempFarm,ncol=3, nrow = 1,
             top = textGrob("Horn, eye and ear temperature per farm, data averaged per round",
                            gp=gpar(fontsize=25,font=1)))
```

Do cow horns have a thermoregulatory function?

Repeated Measures Correlations

```
MeansPerObs <- summaryBy(HornTemp + EyeTemp + EarTemp + TempEnv +
                         HLI + WindSp + Humidity ~ ID_obs,
                       data = Data, FUN = mean, na.rm = T)
MeansPerObs$WindSp.mean[is.na(MeansPerObs$WindSp.mean)] <- 0

Cow_Obs_IDs <- data.frame(Data$ID_obs, Data$CowNr)
Cow_Obs_IDs <- Cow_Obs_IDs %>% distinct(Data.ID_obs, .keep_all = T)
Cow_Obs_IDs <- data.frame(ID_obs = Cow_Obs_IDs$Data.ID_obs,
                          CowNr = Cow_Obs_IDs$Data.CowNr)

MeansPerObs <- merge(MeansPerObs, Cow_Obs_IDs, by = "ID_obs")

RMC_Horn_HLIPerObs <- rmcorr(participant = "CowNr", measure1 = HLI.mean,
                             measure2 = HornTemp.mean, dataset = MeansPerObs)
RMC_Horn_TempEnvPerObs <- rmcorr(participant = "CowNr", measure1 = TempEnv.mean,
                                 measure2 = HornTemp.mean, dataset = MeansPerObs)
RMC_Horn_HumPerObs <- rmcorr(participant = "CowNr", measure1 = Humidity.mean,
                             measure2 = HornTemp.mean, dataset = MeansPerObs)
RMC_Horn_WSPerObs <- rmcorr(participant = "CowNr", measure1 = WindSp.mean,
                            measure2 = HornTemp.mean, dataset = MeansPerObs)

example.rmc.rHLI <- sprintf("%.2f", round(RMC_Horn_HLIPerObs$r, 2))
example.rmc.rTemp <- sprintf("%.2f", round(RMC_Horn_TempEnvPerObs$r, 2))
example.rmc.rH<- sprintf("%.2f", round(RMC_Horn_HumPerObs$r, 2))
example.rmc.rWS <- sprintf("%.2f", round(RMC_Horn_WSPerObs$r, 2))
RMC_Horn_HLIPerObs$p

## [1] 0.0000000000000000000000000000000000000000000000000000000095

RMC_Horn_TempEnvPerObs$p

## [1] 0.000000000000000000000000000000000000000000000000000000000000000000000000000000000067

RMC_Horn_HumPerObs$p

## [1] 0.00000000000015

RMC_Horn_WSPerObs$p

## [1] 0.0000022

RMC_HLI <- ggplot(subset(MeansPerObs, !is.na(HornTemp.mean)),
                  aes(x = HLI.mean, y = HornTemp.mean,
                      group = CowNr,color = CowNr)) +
  geom_point(aes(colour = CowNr)) +
  geom_line(size = 1, aes(y = RMC_Horn_HLIPerObs$model$fitted.values), linetype = 1) +
  theme_bw() +
  scale_color_manual(values = ColPal) +
  labs(x = "Heat load index",
       y = "Horn temperature (°C)",
```

```
       col = "Cow ID") +
  annotate("text", x = 85, y = 16.5, adj = 1, size = 5,
           label = bquote(italic(r[rm])~"="~ .(example.rmc.rHLI))) +
  annotate("text", x = 85, y = 14.5, adj = 1, size = 5,
           label = bquote(italic('p')~"<"~.(0.001))) +
  annotate("text", x = 38, y = 40, adj = 1, size = 7, label = "A") +
  theme(axis.title.y=element_blank(),
        axis.title.x = element_text(size = 12))

RMC_Temp <- ggplot(subset(MeansPerObs, !is.na(HornTemp.mean)),
                   aes(x = TempEnv.mean, y = HornTemp.mean,
                       group = CowNr, color = CowNr)) +
  geom_point(aes(colour = CowNr)) +
  geom_line(size = 1, aes(y = RMC_Horn_TempEnvPerObs$model$fitted.values), linetype = 1) +
  theme_bw() +
  scale_color_manual(values = ColPal)+
  labs(x = "Environmental temperature (°C)",
       y = "Horn temperature (°C)",
       col = "Cow ID") +
  annotate("text", x = 30, y = 16.5, adj = 1, size = 5,
           label = bquote(italic(r[rm])~"="~ .(example.rmc.rTemp))) +
  annotate("text", x = 30, y = 14.5, adj = 1, size = 5,
           label = bquote(italic('p')~"<"~.(0.001))) +
  annotate("text", x = 7, y = 40, adj = 1, size = 7, label = "B") +
  theme(axis.title.y=element_blank(),
        axis.title.x = element_text(size = 12)) +
  guides(col = "none")

RMC_hum <- ggplot(subset(MeansPerObs, !is.na(HornTemp.mean)),
                  aes(x = Humidity.mean, y = HornTemp.mean,
                      group = CowNr, color = CowNr)) +
  geom_point(aes(colour = CowNr)) +
  geom_line(size = 1, aes(y = RMC_Horn_HumPerObs$model$fitted.values), linetype = 1) +
  theme_bw() +
  scale_color_manual(values = ColPal) +
  labs(x = "Humidity (%)",
       y = "Horn temperature (°C)",
       col = "Cow ID") +
  annotate("text", x = 100, y = 16.5, adj = 1, size = 5,
           label = bquote(italic(r[rm])~"="~ .(example.rmc.rH))) +
  annotate("text", x = 100, y = 14.5, adj = 1, size = 5,
           label = bquote(italic('p')~"<"~.(0.001))) +
  annotate("text", x = 23, y = 40, adj = 1, size = 7, label = "C") +
  theme(axis.title.y=element_blank(),
        axis.title.x = element_text(size = 12))+
  guides(col = "none")

RMC_wind <- ggplot(subset(MeansPerObs, !is.na(HornTemp.mean)),
                   aes(x = WindSp.mean, y = HornTemp.mean,
                       group = CowNr, color = CowNr)) +
  geom_point(aes(colour = CowNr)) +
  geom_line(size = 1, aes(y = RMC_Horn_WSPerObs$model$fitted.values), linetype = 1) +
  theme_bw() +
  scale_color_manual(values = ColPal) +
  labs(x = "Wind speed (m/s)",
       y = "Horn temperature (°C)",
       col = "Cow ID") +
```

```
  annotate("text", x = 6.5, y = 16.5, adj = 1, size = 5,
           label = bquote(italic(r[rm])~"="~ .(example.rmc.rWS))) +
  annotate("text", x = 6.5, y = 14.5, adj = 1, size = 5,
           label = bquote(italic('p')~"<"~.(0.001))) +
  annotate("text", x = 0.5, y = 40, adj = 1, size = 7, label = "D") +
  theme(axis.title.y=element_blank(),
        axis.title.x = element_text(size = 12))+
  guides(col = "none")

#Run the following function to get the legend of one of the plots:
legend <- get_legend(RMC_HLI)

grid.arrange(arrangeGrob(RMC_HLI + guides(col = "none"), RMC_Temp,
                         RMC_hum, RMC_wind, nrow = 2, ncol = 2),
             top = textGrob("Repeated measures correlations of horn temperature with environmental \nparameters, averaged per observation",
                            gp = gpar(fontsize = 18, font = 1)),
             left = textGrob("Horn temperature (°C)",
                             gp = gpar(fontsize = 15, font = 1),
                             rot = 90, vjust = 0.5),
             right = legend)
```

Do ear temperatures differ between horned and dehorned cows?

To compare this, it was first checked if the HLI was not significantly different between the farms. For this, farm A and B will be taken together, as these farms both housed horned cows. To correct for differences between individuals, data averaged per round were used.

```
MeansPerRound <- summaryBy(HornTemp + EarTemp + EyeTemp +
                             TempEnv + HLI + WindSp + Humidity ~ ID_round,
                           data = Data, FUN = mean, na.rm = T)

RoundFarm <- data.frame(Data$ID_round, Data$Farm)
RoundFarm <- RoundFarm %>% distinct(Data.ID_round, .keep_all = T)
RoundFarm <- data.frame(ID_round = RoundFarm$Data.ID_round, Farm = RoundFarm$Data.Farm)

MeansPerRound <- merge(MeansPerRound, RoundFarm, by = "ID_round", sort = F, all.x = T)
```

Checking of assumptions:

```
HLI_AB <- MeansPerRound$HLI.mean[MeansPerRound$Farm == "A"|MeansPerRound$Farm == "B"]
HLI_C <- MeansPerRound$HLI.mean[MeansPerRound$Farm == "C"]
shapiro.test(HLI_AB)

##
##  Shapiro-Wilk normality test
##
## data:  HLI_AB
## W = 0.9, p-value = 0.0001

shapiro.test(HLI_C)

##
##  Shapiro-Wilk normality test
##
```

```
## data:   HLI_C
## W = 0.8, p-value = 0.000002

hist(HLI_AB)
hist(HLI_C)
```

A Mann-Whitney U should be used.

Merge farm A and B:

```
MeansPerRound1 <- MeansPerRound
MeansPerRound1[MeansPerRound1 == 'A' | MeansPerRound1 == 'B'] <- 'AB'

wilcox.test(HLI.mean ~ Farm, data=MeansPerRound1, na.rm=TRUE,
            paired=FALSE, exact=FALSE, conf.int=TRUE)

##
##   Wilcoxon rank sum test with continuity correction
##
## data:   HLI.mean by Farm
## W = 2423, p-value = 0.8
## alternative hypothesis: true location shift is not equal to 0
## 95 percent confidence interval:
##   -4.7   3.4
## sample estimates:
## difference in location
##                  -0.67

ggplot(MeansPerRound1, aes(x = Farm, y = HLI.mean, col = Farm))   +
  geom_boxplot(outlier.size=1) +
  labs(x="Farm", y="Heat Load Index",
       title = "Heat Load Index per farm, data averaged per round") +
  theme_bw() +
  guides(col = "none") +
  stat_compare_means(paired = FALSE) +
  stat_n_text() +
  scale_x_discrete(labels=c("A & B", "C"))
```

```
wilcox.test(EarTemp.mean ~ Farm, data=MeansPerRound1, na.rm=TRUE, paired=FALSE, exact=FALSE, conf.int=TRUE)

##
##   Wilcoxon rank sum test with continuity correction
##
## data:   EarTemp.mean by Farm
## W = 2175, p-value = 0.2
## alternative hypothesis: true location shift is not equal to 0
## 95 percent confidence interval:
##   -1.15   0.25
## sample estimates:
## difference in location
##                  -0.43

ggplot(MeansPerRound1, aes(x = Farm, y = EarTemp.mean, col = Farm))   +
  geom_boxplot(outlier.size=1) +
  labs(x="Farm", y="Ear temperature (°C)") +
  theme_bw() +
```

```
  guides(col = "none") +
  coord_cartesian(ylim=c(14, 40)) +
  stat_compare_means(paired = FALSE, label.y= 15) +
  scale_x_discrete(labels=c("A & B", "C"))
```

Does eye temperature differ between horned and dehorned cows?

```
wilcox.test(EyeTemp.mean ~ Farm, data=MeansPerRound1, na.rm=TRUE,
            paired=FALSE, exact=FALSE, conf.int=TRUE)

##
##  Wilcoxon rank sum test with continuity correction
##
## data:  EyeTemp.mean by Farm
## W = 1021, p-value = 0.000000004
## alternative hypothesis: true location shift is not equal to 0
## 95 percent confidence interval:
##  -0.79 -0.39
## sample estimates:
## difference in location
##                  -0.59

ggplot(MeansPerRound1, aes(x = Farm, y = EyeTemp.mean, col = Farm))  +
  geom_boxplot(outlier.size=1) +
  labs(x="Farm", y="Eye temperature (°C)") +
  theme_bw() +
  guides(col = "none") +
  stat_compare_means(paired = FALSE, label.y = 30) +
  coord_cartesian(ylim=c(30, 40)) +
  scale_x_discrete(labels=c("A & B", "C"))
```

Is cow horn temperature influenced by behaviour?

```
Data1 <- Data
Data1$Non_social <- as.character(Data1$Non_social)
Data1$Non_social[Data1$Non_social == "Being alert" |
                 Data1$Non_social == "Milking" |
                 Data1$Non_social == "Drinking" |
                 Data1$Non_social == "None"] <- "Other"

Data1$Social <- as.character(Data1$Social)
Data1$Social[Data1$Social == "Allogrooming donor" |
             Data1$Social == "Allogrooming recipient" |
             Data1$Social == "Head resting" |
             Data1$Social == "Playing"] <- "Yes"
Data1$Social[Data1$Social == "None"] <- "No"
```

To avoid pseudoreplication, two dataframes were made with averaged horn temperatures per round, for each non-social or social duration behaviour.

```
MeansRoundNon_soc <- summaryBy(HornTemp + TempEnv + HLI +
                               WindSp + Humidity ~ ID_round * Non_social,
                              data = Data1, FUN = mean, na.rm = T)
# Adjust the levels of Non_social so that 'Other' is last:
```

```
MeansRoundNon_soc$Non_social <- factor(MeansRoundNon_soc$Non_social,
                                       levels = c("Feeding","Ruminating","Self-grooming","Other"))

MeansRoundSoc <- summaryBy(HornTemp + TempEnv + HLI +
                           WindSp + Humidity ~ ID_round * Social,
                         data = Data1, FUN = mean, na.rm = T)

ggplot(aes(x = Non_social, y = HornTemp.mean), data = MeansRoundNon_soc) +
  geom_boxplot() +
  labs(x = "Behavioural category",
       y = "Horn temperature (ºC)",
       title = "Horn temperature for different non-social behavioural \ncategories, averaged per round") +
  theme_bw(base_size = 20) +
  stat_n_text()

ggplot(aes(x = Social, y = HornTemp.mean), data = MeansRoundSoc) +
  geom_boxplot() +
  labs(x = "Social behaviours?",
       y = "Horn temperature (ºC)",
       title = "Horn temperature for different social behavioural \ncategories, averaged per round") +
  theme_bw(base_size = 20) +
  stat_n_text()
```

```
anovaHT_NS <- lm(HornTemp.mean ~ Non_social, data = MeansRoundNon_soc)
summary(anovaHT_NS)

##
## Call:
## lm(formula = HornTemp.mean ~ Non_social, data = MeansRoundNon_soc)
##
## Residuals:
##     Min      1Q  Median      3Q     Max
## -18.869  -1.904   0.715   2.291   7.522
##
## Coefficients:
##                         Estimate Std. Error t value            Pr(>|t|)
## (Intercept)              31.9444     0.3896   82.00 <0.0000000000000002 ***
## Non_socialRuminating      0.6588     0.5951    1.11                0.27
## Non_socialSelf-grooming  -0.0781     0.8875   -0.09                0.93
## Non_socialOther           0.0233     0.5367    0.04                0.97
## ---
## Signif. codes:  0 '***' 0.001 '**' 0.01 '*' 0.05 '.' 0.1 ' ' 1
##
## Residual standard error: 3.6 on 269 degrees of freedom
##   (152 observations deleted due to missingness)
## Multiple R-squared:  0.006,  Adjusted R-squared:  -0.00508
## F-statistic: 0.541 on 3 and 269 DF,  p-value: 0.654

par(mfrow = c(2,2)) # first create a 2x2 matrix of plots
plot(anovaHT_NS)
```

Assumptions were not met, so Kruskal-Wallis was used:

```
kruskal.test(HornTemp.mean ~ Non_social, data = MeansRoundNon_soc)
```

```
##
##     Kruskal-Wallis rank sum test
##
## data:   HornTemp.mean by Non_social
## Kruskal-Wallis chi-squared = 2, df = 3, p-value = 0.6

HornTempSocNo <- MeansRoundSoc$HornTemp.mean[MeansRoundSoc$Social == "No"]
HornTempSocYes <- MeansRoundSoc$HornTemp.mean[MeansRoundSoc$Social == "Yes"]
shapiro.test(HornTempSocNo)

##
##     Shapiro-Wilk normality test
##
## data:   HornTempSocNo
## W = 0.9, p-value = 0.0000008

shapiro.test(HornTempSocYes)

##
##     Shapiro-Wilk normality test
##
## data:   HornTempSocYes
## W = 0.8, p-value = 0.01

hist(HornTempSocNo)
hist(HornTempSocYes)
```

```
wilcox.test(HornTemp.mean ~ Social, data=MeansRoundSoc, na.rm=TRUE,
            paired=FALSE, exact=FALSE, conf.int=TRUE)

##
##     Wilcoxon rank sum test with continuity correction
##
## data:   HornTemp.mean by Social
## W = 584, p-value = 0.7
## alternative hypothesis: true location shift is not equal to 0
## 95 percent confidence interval:
##   -1.5   1.8
## sample estimates:
## difference in location
##                    0.3
```

Do horn temperatures change during a rumination bout?

```
DataRum <- read_excel("Data Ruminating.xlsx")

ggplot(DataRum, aes(x = Time, y = cHorn)) +
  geom_point(shape = 16,
             size = 3) +
  labs(x="Time (minutes)", y="Relative horn temperature (°C)",
       title = "All temperature observations (n=42 cows), standardized \nfor starting temperature (°C)") +
  theme_bw(base_size = 20) +
  guides(col = "none") +
  geom_hline(yintercept = 0, color = "red", size = 1)

t20 <- subset(DataRum, Location == 1 & Time <= 20)
```

Now fit a linear mixed-effects model:

```
fit=lmer(Horn ~ factor(Time) + factor(Farm) +
           cut(Windspeed, c(-1, 1.3, 2.5, 3.1, 7.6)) +
           cut(THI, c(51, 56, 60, 64, 68, 73)) +
           cut(Parity, c(0,2,4,6,10)) + (1|Cownr),
         data=t20, REML = F)
summary(fit)

## Linear mixed model fit by maximum likelihood  ['lmerMod']
## Formula: Horn ~ factor(Time) + factor(Farm) + cut(Windspeed, c(-1, 1.3,
##     2.5, 3.1, 7.6)) + cut(THI, c(51, 56, 60, 64, 68, 73)) + cut(Parity,
##     c(0, 2, 4, 6, 10)) + (1 | Cownr)
##    Data: t20
##
##      AIC      BIC   logLik deviance df.resid
##     1954     2084     -948     1896      623
##
## Scaled residuals:
##    Min     1Q Median     3Q    Max
## -3.617 -0.579  0.005  0.541  4.267
##
## Random effects:
##  Groups   Name        Variance Std.Dev.
##  Cownr    (Intercept) 8.351    2.890
##  Residual             0.777    0.881
## Number of obs: 652, groups:  Cownr, 41
##
## Fixed effects:
##                                                Estimate Std. Error t value
## (Intercept)                                    28.30999    1.51976   18.63
## factor(Time)1                                  -0.16098    0.19464   -0.83
## factor(Time)2                                  -0.26220    0.19464   -1.35
## factor(Time)3                                   0.00976    0.19464    0.05
## factor(Time)4                                  -0.02683    0.19464   -0.14
## factor(Time)5                                  -0.16341    0.19464   -0.84
## factor(Time)6                                   0.14467    0.19485    0.74
## factor(Time)7                                   0.03858    0.19485    0.20
## factor(Time)8                                  -0.04923    0.19485   -0.25
## factor(Time)9                                   0.02760    0.19485    0.14
## factor(Time)10                                  0.06784    0.19485    0.35
## factor(Time)12                                  0.08723    0.19615    0.44
## factor(Time)14                                 -0.10462    0.19648   -0.53
## factor(Time)16                                  0.06602    0.19647    0.34
## factor(Time)18                                 -0.22379    0.19517   -1.15
## factor(Time)20                                 -0.21326    0.20715   -1.03
## factor(Farm)1                                  -1.21157    1.22876   -0.99
## cut(Windspeed, c(-1, 1.3, 2.5, 3.1, 7.6))(1.3,2.5] -0.33838    0.34529   -0.98
## cut(Windspeed, c(-1, 1.3, 2.5, 3.1, 7.6))(2.5,3.1] -0.98617    0.34710   -2.84
## cut(Windspeed, c(-1, 1.3, 2.5, 3.1, 7.6))(3.1,7.6] -0.46999    0.43914   -1.07
## cut(THI, c(51, 56, 60, 64, 68, 73))(56,60]     2.35718    0.42848    5.50
## cut(THI, c(51, 56, 60, 64, 68, 73))(60,64]     4.31912    0.84670    5.10
## cut(THI, c(51, 56, 60, 64, 68, 73))(64,68]     4.87184    1.02029    4.77
## cut(THI, c(51, 56, 60, 64, 68, 73))(68,73]     5.65990    1.10479    5.12
## cut(Parity, c(0, 2, 4, 6, 10))(2,4]           -0.43717    0.97342   -0.45
## cut(Parity, c(0, 2, 4, 6, 10))(4,6]            0.69140    2.20124    0.31
## cut(Parity, c(0, 2, 4, 6, 10))(6,10]           2.57190    3.01655    0.85
```

```
##
## Correlation matrix not shown by default, as p = 27 > 12.
## Use print(x, correlation=TRUE)  or
##     vcov(x)        if you need it

drop1(fit, test = "Chisq")

## Single term deletions
##
## Model:
## Horn ~ factor(Time) + factor(Farm) + cut(Windspeed, c(-1, 1.3,
##     2.5, 3.1, 7.6)) + cut(THI, c(51, 56, 60, 64, 68, 73)) + cut(Parity,
##     c(0, 2, 4, 6, 10)) + (1 | Cownr)
##                                       npar  AIC  LRT     Pr(Chi)
## <none>                                     1955
## factor(Time)                            15 1937 12.0       0.679
## factor(Farm)                             1 1953  1.0       0.326
## cut(Windspeed, c(-1, 1.3, 2.5, 3.1, 7.6))   3 1959 10.9       0.012 *
## cut(THI, c(51, 56, 60, 64, 68, 73))        4 1983 36.6 0.00000022 ***
## cut(Parity, c(0, 2, 4, 6, 10))             3 1950  1.2       0.754
## ---
## Signif. codes:  0 '***' 0.001 '**' 0.01 '*' 0.05 '.' 0.1 ' ' 1
```

Since ‘parity’ had a low AIC, it was dropped from the model:

```
fit=lmer(Horn ~ factor(Time) + factor(Farm) +
           cut(Windspeed, c(-1, 1.3, 2.5, 3.1, 7.6)) +
           cut(THI, c(51, 56, 60, 64, 68, 73)) + (1|Cownr),
         data=t20, REML = F)
summary(fit)

## Linear mixed model fit by maximum likelihood  ['lmerMod']
## Formula: Horn ~ factor(Time) + factor(Farm) + cut(Windspeed, c(-1, 1.3,
##     2.5, 3.1, 7.6)) + cut(THI, c(51, 56, 60, 64, 68, 73)) + (1 |      Cownr)
##    Data: t20
##
##      AIC      BIC   logLik deviance df.resid
##     1950     2066     -949     1898      626
##
## Scaled residuals:
##     Min     1Q Median     3Q    Max
## -3.621 -0.580  0.007  0.543  4.266
##
## Random effects:
##  Groups   Name        Variance Std.Dev.
##  Cownr    (Intercept) 8.681    2.946
##  Residual             0.776    0.881
## Number of obs: 652, groups:  Cownr, 41
##
## Fixed effects:
##                                          Estimate Std. Error t value
## (Intercept)                              28.16197    1.36168   20.68
## factor(Time)1                            -0.16098    0.19458   -0.83
## factor(Time)2                            -0.26220    0.19458   -1.35
## factor(Time)3                             0.00976    0.19458    0.05
## factor(Time)4                            -0.02683    0.19458   -0.14
## factor(Time)5                            -0.16341    0.19458   -0.84
```

```
## factor(Time)6                                          0.14446   0.19479    0.74
## factor(Time)7                                          0.03837   0.19479    0.20
## factor(Time)8                                         -0.04944   0.19479   -0.25
## factor(Time)9                                          0.02739   0.19479    0.14
## factor(Time)10                                         0.06763   0.19479    0.35
## factor(Time)12                                         0.08702   0.19608    0.44
## factor(Time)14                                        -0.10427   0.19642   -0.53
## factor(Time)16                                         0.06630   0.19641    0.34
## factor(Time)18                                        -0.22352   0.19511   -1.15
## factor(Time)20                                        -0.21315   0.20709   -1.03
## factor(Farm)1                                         -1.08408   1.23177   -0.88
## cut(Windspeed, c(-1, 1.3, 2.5, 3.1, 7.6))(1.3,2.5] -0.32647   0.34402   -0.95
## cut(Windspeed, c(-1, 1.3, 2.5, 3.1, 7.6))(2.5,3.1] -0.97008   0.34669   -2.80
## cut(Windspeed, c(-1, 1.3, 2.5, 3.1, 7.6))(3.1,7.6] -0.44947   0.43789   -1.03
## cut(THI, c(51, 56, 60, 64, 68, 73))(56,60]          2.33751   0.42571    5.49
## cut(THI, c(51, 56, 60, 64, 68, 73))(60,64]          4.24783   0.83343    5.10
## cut(THI, c(51, 56, 60, 64, 68, 73))(64,68]          4.78703   1.00009    4.79
## cut(THI, c(51, 56, 60, 64, 68, 73))(68,73]          5.57398   1.08514    5.14

##
## Correlation matrix not shown by default, as p = 24 > 12.
## Use print(x, correlation=TRUE)    or
##     vcov(x)        if you need it
```

```
drop1(fit, test = "Chisq")
```

```
## Single term deletions
##
## Model:
## Horn ~ factor(Time) + factor(Farm) + cut(Windspeed, c(-1, 1.3,
##     2.5, 3.1, 7.6)) + cut(THI, c(51, 56, 60, 64, 68, 73)) + (1 |
##     Cownr)
##                                              npar  AIC  LRT    Pr(Chi)
## <none>                                            1950
## factor(Time)                                   15 1932 12.0      0.680
## factor(Farm)                                    1 1948  0.8      0.380
## cut(Windspeed, c(-1, 1.3, 2.5, 3.1, 7.6))      3 1954 10.7      0.013 *
## cut(THI, c(51, 56, 60, 64, 68, 73))            4 1979 36.8 0.0000002 ***
## ---
## Signif. codes:  0 '***' 0.001 '**' 0.01 '*' 0.05 '.' 0.1 ' ' 1
```

```
confint(fit)
```

```
##                  2.5 % 97.5 %
## .sig01            2.36   3.79
## .sigma            0.83   0.93
## (Intercept)      25.48  31.02
## factor(Time)1    -0.54   0.22
## factor(Time)2    -0.64   0.12
## factor(Time)3    -0.37   0.39
## factor(Time)4    -0.41   0.36
## factor(Time)5    -0.55   0.22
## factor(Time)6    -0.24   0.53
## factor(Time)7    -0.34   0.42
## factor(Time)8    -0.43   0.33
## factor(Time)9    -0.35   0.41
## factor(Time)10   -0.31   0.45
## factor(Time)12   -0.30   0.47
```

```
## factor(Time)14                                      -0.49     0.28
## factor(Time)16                                      -0.32     0.45
## factor(Time)18                                      -0.61     0.16
## factor(Time)20                                      -0.62     0.19
## factor(Farm)1                                       -3.65     1.34
## cut(Windspeed, c(-1, 1.3, 2.5, 3.1, 7.6))(1.3,2.5] -1.00     0.35
## cut(Windspeed, c(-1, 1.3, 2.5, 3.1, 7.6))(2.5,3.1] -1.65    -0.29
## cut(Windspeed, c(-1, 1.3, 2.5, 3.1, 7.6))(3.1,7.6] -1.31     0.41
## cut(THI, c(51, 56, 60, 64, 68, 73))(56,60]          1.48     3.19
## cut(THI, c(51, 56, 60, 64, 68, 73))(60,64]          2.50     5.94
## cut(THI, c(51, 56, 60, 64, 68, 73))(64,68]          2.64     6.81
## cut(THI, c(51, 56, 60, 64, 68, 73))(68,73]          3.27     7.76
```

Script S3: Reliability calculations

R script to calculate the observer reliability for scoring of the IRT images

To compare whether observer 1 and 2 analysed the thermal photos the same (inter-observer reliability), and whether both observers analysed the photos the same at the end of the analyses as at the beginning (inter-observer reliability) an Intraclass Correlation Coefficient can be calculated.

```
Obs_reliability_IRT <-
  as.data.frame(read_excel("Example data/Reliability example 1.xlsx"))

library("irr")

## Loading required package: lpSolve

icc(Obs_reliability_IRT, model = "oneway", type = "consistency", unit = "single")

##   Single Score Intraclass Correlation
##
##     Model: oneway
##     Type : consistency
##
##     Subjects = 252
##       Raters = 2
##       ICC(1) = 0.966
##
##   F-Test, H0: r0 = 0 ; H1: r0 > 0
##   F(251,252) = 58.4 , p = 8.74e-151
##
##   95%-Confidence Interval for ICC Population Values:
##    0.957 < ICC < 0.974
```

Kappa coefficient calculations

The kappa coefficient was calculated by comparing, for each used observation, the score sheets from both observers and filling in an agreement/disagreement matrix. The matrix calculated the proportion of agreement (Po) and the chance of agreement (Pc). Then, the following equation was used:

$$k = (Po - Pc)/(1 - Pc)$$

The confidence interval for the kappa coefficient of each observation was calculated as well, using the following equation [89]:

$$k - 1.96 * SE_k \ to \ k + 1.96 * SE_k$$

Where k is Cohen's kappa, 1.96 is the constant used when a CI of 95% is desired, and SE_k is the standard error of k, calculated with this formula [89]:

$$SE_K = \sqrt{\frac{Po(1 - Po)}{(1 - Pc)^2}}$$

Then, the mean of the kappa coefficient of the used observations each time was calculated using the AVERAGE() function of Excel. The confidence interval of this mean was calculated using this equation [89]:

$$\bar{X} - 1.96 * \left(\frac{s}{\sqrt{n}}\right) \ to \ \bar{X} + 1.96 * \left(\frac{s}{\sqrt{n}}\right)$$

Where $\bar{X}$ is the sample mean, 1.96 the constant used for the 95% CI, s is the sample standard deviation and n is the sample size.

Results for reliability calculations

Table S2: Overview of the ICC coefficients for the intra- and inter-observer reliability. For each ICC calculation, the intraclass correlation coefficient (ICC), definition, 95% confidence interval (95% CI), F-value, p-value and the percentage of images that were in- or excluded in agreement between both observers are reported.

	When	What	ICC	Definition	95% CI	F-value	p-value	% images scored in agreement
**Intra-observer reliability**	End farm A	End vs middle farm A – obs. 1	0.999	Con-sistency	0.997 – 0.999	(23,24) = 1415	<0.001	78.72%
	End farm A	End vs middle farm A – obs. 2	0.983	Con-sistency	0.957 – 0.994	(17,18) = 118	<0.001	64.18%
	End farm B	End vs middle farm B – obs. 1	0.994	Con-sistency	0.987 – 0.997	(26,27) = 335	<0.001	85.12%
**Inter-observer reliability**	Beginning farm A	Obs. 1 vs Obs. 2	0.972	Con-sistency	0.945 – 0.986	(32,33) = 71.1	<0.001	89.29%
	End farm A	Obs. 1 vs Obs. 2	0.980	Con-sistency	0.964 – 0.989	(41,42) = 101	<0.001	67.80%
	Beginning farm B	Obs. 1 vs Obs. 2	0.867	Con-sistency	0.762 – 0.928	(38,39) = 14	<0.001	84.33%

Table S3. Horn temperature during rumination. Estimates and confidence intervals of the linear mixed-effects model testing horn temperature during the 20 min rumination bouts (N = 41 cows).

Predictor	Estimate	Confidence intervals	
		**2.5%**	**97.5%**
(Intercept)	28.16	25.48	31.02
Time 1	-0.16	-0.54	0.22
Time 2	-0.26	-0.64	0.12
Time 3	0.01	-0.37	0.39
Time 4	-0.03	-0.41	0.36
Time 5	-0.16	-0.54	0.22
Time 5	-0.16	-0.54	0.22
Time 6	0.14	-0.24	0.53
Time 7	0.04	-0.34	0.42
Time 8	-0.05	-0.43	0.33
Time 9	0.03	-0.35	0.41
Time 10	0.07	-0.31	0.45
Time 12	0.09	-0.30	0.47
Time 14	-0.10	-0.49	0.28
Time 16	0.07	-0.32	0.45
Time 18	-0.22	-0.61	0.16
Time 20	-0.21	-0.62	0.19
Farm B	-1.08	-3.65	1.34
Windspeed (1.3; 2.5]	-0.33	-1.00	0.35
Windspeed (2.5; 3.1]	-0.97	-1.65	-0.29
Windspeed (3.1; 7.6]	-0.45	-1.31	0.41
THI-score (56, 60]	2.34	1.48	3.19
THI-score (60, 64]	4.25	2.50	5.94
THI-score (64, 68]	4.79	2.64	6.80
THI-score (68, 73]	5.57	3.27	7.76

References

35. Byrne, D.T.; Berry, D.P.; Esmonde, H.; McHugh, N. Temporal, Spatial, Inter-, and Intra-Cow Repeatability of Thermal Imaging1. *J. Anim. Sci.* **2017**, *95*, 970–979, doi:10.2527/jas.2016.1005.
37. Cuthbertson, H.; Tarr, G.; Loudon, K.; Lomax, S.; White, P.; McGreevy, P.; Polkinghorne, R.; González, L.A. Using Infrared Thermography on Farm of Origin to Predict Meat Quality and Physiological Response in Cattle (Bos Taurus) Exposed to Transport and Marketing. *Meat Sci.* **2020**, *169*, 108173, doi:10.1016/j.meatsci.2020.108173.
38. Macmillan, K.; Colazo, M.G.; Cook, N.J. Evaluation of Infrared Thermography Compared to Rectal Temperature to Identify Illness in Early Postpartum Dairy Cows. *Res. Vet. Sci.* **2019**, *125*, 315–322, doi:10.1016/j.rvsc.2019.07.017.
39. Martello, L.S.; da Luz e Silva, S.; da Costa Gomes, R.; da Silva Corte, R.R.P.; Leme, P.R. Infrared Thermography as a Tool to Evaluate Body Surface Temperature and Its Relationship with Feed Efficiency in Bos Indicus Cattle in Tropical Conditions. *Int. J. Biometeorol.* **2016**, *60*, 173–181, doi:10.1007/s00484-015-1015-9.
40. Talukder, S.; Kerrisk, K.L.; Ingenhoff, L.; Thomson, P.C.; Garcia, S.C.; Celi, P. Infrared Technology for Estrus Detection and as a Predictor of Time of Ovulation in Dairy Cows in a Pasture-Based System. *Theriogenology* **2014**, *81*, 925–935, doi:10.1016/j.theriogenology.2014.01.009.
41. Uddin, J.; McNeill, D.M.; Lisle, A.T.; Phillips, C.J.C. A Sampling Strategy for the Determination of Infrared Temperature of Relevant External Body Surfaces of Dairy Cows. *Int. J. Biometeorol.* **2020**, *64*, 1583–1592, doi:10.1007/s00484-020-01939-4.
42. Franchi, G.A.; Jensen, M.B.; Herskin, M.S.; McNeill, D.M.; Phillips, C.J.C. Assessing Response to Dry-off in Dairy Cows Kept Outdoors Using Spontaneous Behaviours and Infrared Thermography—a Pilot Study. *Trop. Anim. Health Prod.* **2021**, *53*, 1–4, doi:10.1007/s11250-020-02487-0.
43. Uddin, J.; Phillips, C.J.C.; Goma, A.A.; McNeill, D.M. Relationships between Infrared Temperature and Laterality. *Appl. Anim. Behav. Sci.* **2019**, *220*, 104855, doi:10.1016/j.applanim.2019.104855.
44. Cuthbertson, H.; Tarr, G.; González, L.A. Methodology for Data Processing and Analysis Techniques of Infrared Video Thermography Used to Measure Cattle Temperature in Real Time. *Comput. Electron. Agric.* **2019**, *167*, 105019, doi:10.1016/j.compag.2019.105019.
89. McHugh, M.L. Interrater Reliability: The Kappa Statistic. *Biochem. Medica* **2012**, *22*, 276.
